# Challenges to undertaking randomised trials with looked after children in social care settings

**DOI:** 10.1186/s13063-015-0708-z

**Published:** 2015-05-07

**Authors:** Gillian Mezey, Fiona Robinson, Rona Campbell, Steve Gillard, Geraldine Macdonald, Deborah Meyer, Chris Bonell, Sarah White

**Affiliations:** Population Health Research Institute (PHRI), St Georges University of London, Cranmer Terrace, London, SW17 ORE England; School of Social and Community Medicine, Bristol University, Canynge Hall, 39 Whatley Road, Bristol, BS8 2PS England; School of Sociology, Social Policy and Social Work, Queen’s University Belfast, 6 College Park, Belfast, BT7 1LP Ireland; Social Science Research Unit, Faculty of Children and Learning, Institute of Education 20 Bedford Way, London, WC1H 0AL England

**Keywords:** Trials, Local authorities, Looked after children

## Abstract

**Background:**

Randomised controlled trials (RCTs) are widely viewed as the gold standard for assessing effectiveness in health research; however many researchers and practitioners believe that RCTs are inappropriate and un-doable in social care settings, particularly in relation to looked after children. The aim of this article is to describe the challenges faced in conducting a pilot study and phase II RCT of a peer mentoring intervention to reduce teenage pregnancy in looked after children in a social care setting.

**Methods:**

Interviews were undertaken with social care professionals and looked after children, and a survey conducted with looked after children, to establish the feasibility and acceptability of the intervention and research design.

**Results:**

Barriers to recruitment and in managing the intervention were identified, including social workers acting as informal gatekeepers; social workers concerns and misconceptions about the recruitment criteria and the need for and purpose of randomisation; resource limitations, which made it difficult to prioritise research over other demands on their time and difficulties in engaging and retaining looked after children in the study.

**Conclusions:**

The relative absence of a research infrastructure and culture in social care and the lack of research support funding available for social care agencies, compared to health organisations, has implications for increasing evidence-based practice in social care settings, particularly in this very vulnerable group of young people.

**Electronic supplementary material:**

The online version of this article (doi:10.1186/s13063-015-0708-z) contains supplementary material, which is available to authorized users.

## Background

In recent years, there have been calls for a more evidence-based practice (EBP) approach within social care. However, it has been suggested that there is a fundamental incompatibility between social work and the science of EBP as conceptualised within health [1-5].

Randomised controlled trials (RCTs) are widely considered to be the gold standard for assessing effectiveness in health and clinical based research [6]. However RCTs within the field of social care in the UK are uncommon [7,8].

Opposition to RCTs by social care professionals has ostensibly been based around feasibility, epistemology and ethics, particularly in relation to the use of randomisation [5,9], although similar concerns have also been reported by those working in health [10-12] and education [13,14]. The rationale for using RCT methodology is ‘equipoise’, i.e. uncertainty as to whether the intervention condition is beneficial in comparison to the control condition. Randomisation to the non-intervention armis often perceived as the withholding of potentially beneficial treatment and may trigger a gatekeeping effect by social workers, thereby denying eligible populations the opportunity to participate [7,15]. Irrespective of the context, recruitment difficulties in RCTs can result in significant delays to the study timeline, increased costs and a loss of statistical power if the required sample size is not achieved [16,17]. Such problems can be exacerbated in settings where there is ambivalence towards or limited organisational experience of trials.

Research and Development in social work receives a far smaller share of total spend, compared with health [18]. Furthermore, NHS Trusts are required to support the costs of providing an experimental intervention and commissioners are committed to providing the funding to support National Institute for Health Research (NIHR) funded research (updating and strengthening previous similar agreements). No such culture or provision exists in social care [15,18,19].

Compared with health professionals, social workers receive minimal training in research methodology or critical appraisal and there is considerable disagreement as to what constitutes best practice with regard to research participation and conduct, with many reporting a preference for ‘practice wisdom’ or intuition [2,3,18].

This article draws on the experience of conducting an RCT in a UK social care context to explore these and other barriers to the successful implementation of randomised trials in social care, specifically with a hard-to-reach population of looked after children (LAC). LAC are children in the UK who are in the care of the state or are recent care leavers. They are a highly vulnerable population, who are more likely to have experienced several risk factors for social exclusion [20-29]; they are at greater risk of disengaging from education, truancy and school exclusion [30]; they have higher rates of mental health problems [31,32] and are around three times more likely to run away or go missing, compared with children living in private households [33,34].

There is some evidence to suggest that accessing LAC for research purposes can be particularly difficult, both because of concerns about their vulnerability and because of the many professionals and carers surrounding them whose responsibility it is to protect them from further harm, including excessive or unwarranted approaches by researchers [35,36]. This article builds on that evidence and considers the organisational, cultural and infrastructure implications for conducting future trials with LAC and in social care more generally.

## Method

### Setting

The study was commissioned by the NIHR’s Health Technology Assessment Programme and comprised the development and piloting of a peer-mentoring intervention to reduce teenage pregnancy rates in LAC, followed by a phase II RCT of the intervention. The peer mentors were young women, aged 19–25, who had themselves been looked after; they provided individual mentoring support to a female LAC aged 14–18 for up to 1 year. Mentors received a 3 day training programme and were provided with a monthly support group facilitated by a Local Authority (LA) project coordinator (PC).

### Ethical approval

Ethical approval was granted in December 2010 by the Research and Ethics Committee based at the London School of Hygiene and Tropical Medicine (reference no. 5866). LSHTM was approached, as St Georges University had no Research and Ethics committee. Two of the applicants were at the time working at LSHTM. Moreover, this committee and the institution had a great deal of experience and a track record in considering ethical issues related to social science-based, rather than medical-based, research.

### Setting and recruitment

The trial was conducted in two London local authorities (Las, referred to as LA1 and LA2) and one non-London LA (LA3), and the recruitment strategy was as follows: Directors of Children’s Services in the participating LAs agreed to release a senior social worker to be the local project co-ordinator (PC) on the project, with an estimated time commitment of around 3 hours a week. PCs were required to lead on the identification and recruitment of mentors and mentees, to manage the project at a local level, to provide LA social workers with preliminary information about the study and invite them to identify potential candidates. LAC aged 14–17 were to be identified as potential mentees and young people, aged 18 to 24, who had left care were to be recruited as peer mentors. The social workers were asked to speak with potential participants about the study, before passing their details on to the PC and the research team. If they expressed an interest, the young people were then contacted by the researchers,to arrange a meeting. Informed verbal and written consent was obtained from all participants, prior to completing the baseline interview.

All potential mentors and mentees were provided with information sheets, explaining the study, including for mentees the process and purpose of randomisation. Young people under age 16 were invited to have their social worker or other LA individual present when obtaining consent. If they preferred to attend alone, the researchers spoke to their social worker to confirm their capacity to consent. Young people aged between 16 and 18 could also elect to have a third person present if they wished. Fraser guidelines [37], which set out criteria for determining if a child is mature enough to make decisions, specifically around contraception and sexual matters, were followed. A copy of the mentee consent form was sent to the mentee’s social worker together with details of the PC.

Mentors and mentees were advised to direct initial queries about the research to the researchers. However, concerns or problems experienced in relation to the day-to-day mentoring role, such as practicalities of setting up mentoring appointments, concerns about the mentee or the relationship, or safeguarding issues were to be directed, in the first instance, to the PC, who could in turn communicate this to the relevant social worker for the young person or to the research team. Mentees who were concerned about the conduct of their mentor, or were unhappy about their relationship with their mentor, were advised to contact the PC or their social worker directly.

We developed protocols for dealing with a disclosure of significant risk or on-going harm involving a mentor or mentored young person. Prior to giving consent, all participants were informed of the limits of confidentiality in research interviews, and were told that their social worker or another member of their care network would be informed if any such disclosures were made.

*Randomisation* LAC mentees participating in the RCT were individually randomised, with half receiving the peer mentor intervention and half ‘usual care’, consisting of the services already available to them due to their status as LAC [38].

In neither the pilot nor the RCT was the recruitment target met, even with an extension of the recruitment period from 6 weeks to 12 weeks. Four mentor-mentee pairs were recruited and commenced pilot intervention, instead of the target number of six.

### Barriers to recruitment and challenges to implementation

In order to explore barriers to recruitment and the acceptability of implementing the trial, we conducted 13 semi-structured interviews with PCs, senior managers and social workers involved in the pilot intervention and the trial. The interviews included topics about respondents’ experiences of recruiting young people to the study, difficulties encountered and possible solutions to those difficulties. Five focus groups were also conducted with a total of 21 social workers, health and education professionals from the three participating LAs.

Semi-structured interviews were conducted with 19 mentees and 13 mentors at the end of the intervention period, exploring their views on the research and randomisation. In addition, LAC and care leavers nationally were invited to respond to an online survey advertised in the Who Cares? Trust (a national charity for LAC) magazine (see Additional file 1). This was undertaken to boost our understanding of young people’s views on recruitment, randomisation and participation in research by including young people who were not already participating in the study. Sixty four responses were received (27 from LAC aged 14–18, 37 from care leavers aged 19–25; mean age 19.4 years, SD 3.1).

### Lessons from the Pilot and Modifications Made to the Intervention for the RCT

Interviews were conducted with mentors, mentees, social workers in LA1 and the PC in LA1 after 3 months and prior to commencing recruitment for the RCT, in order to explore the reasons for and to rectify problems around recruitment of mentors and mentees for the phase 11 trial. The main problems that were identified were: not adhering to eligibility criteria; attrition of mentors due to delays in implementation of the intervention; absence of ring-fenced time for PCs to support the study; difficulties in establishing early and regular meetings between mentors and mentees.

Despite the fact that the only recruitment criteria to have been stipulated by the team were that young people should be looked after and aged 14–18,in our interview with the PC involved with the pilot we were made aware that social workers were preferentially recruiting vulnerable, disengaged young women who were ‘more at risk of becoming pregnant’ (LA1 SM). The recruitment criteria were therefore re-drafted for the RCT and distributed to the PCs and social workers in the participating LAs, re-emphasising the inclusion criteria and expectations around the mentor, mentee and PC roles and responsibilities. The research team also became more proactive in the recruitment process, including weekly telephone calls to the PCs, to check on recruitment progress and troubleshoot any difficulties. Finally, in order to increase the pool of potential candidates and following advice from PCs, other professionals involved with LAC, e.g. health and education professionals, were sent information about the study and asked to nominate young people for the intervention.

In the pilot, mentor recruitment had taken place before mentee recruitment, largely because of the need to arrange training dates and venues in advance. This meant that mentors who had received training and were keen to start had to wait several months to be allocated a mentee. The delay in starting the intervention had resulted in a loss of momentum and enthusiasm by mentors involved in the pilot; it was therefore stipulated that mentor and mentee recruitment had to take place concurrently for the RCT. Interviews with social workers and the PC involved in the pilot suggested that concurrent recruitment should be achievable.

In the pilot, the PC only organised one support meeting for mentors during the first 3 months of the pilot, even though support meetings were meant to have been held monthly and only one of the four mentors attended this meeting. However, when we interviewed the pilot mentors, they all stated that they wanted and needed more support to carry out their role. We therefore asked PCs to ensure that the monthly meetings took place every month during the trial and that mentors were able to contact them outside these meetings on an ad hoc basis, for support, or if they encountered any problems.

The PC involved in the pilot had said that she had found it difficult to carry out her role in full because of other competing work commitments. She felt that, without dedicated time to devote to the study, Project Coordinators involved in the RCT would struggle to carry out all the tasks required of them. As a result of this feedback, the PC in one of the Local Authorities was given some dedicated time for the RCT, whilst in the two other Local Authorities, the PC role ended up being allocated to two individuals in an attempt to lessen the work load involved.

In the pilot, mentors had reported finding it difficult to set up the first meeting with their mentees. Therefore, PCs were asked to attend the first meeting between the mentor and mentee in the RCT. PCs were also asked in their monthly support meetings with mentors to reiterate the expectations of the study and the intervention, including the need to have at least one face-to-face meeting with their mentee every week and to complete a contact diary. All mentors received payment in the form of shopping vouchers in recognition of their role and to cover activities they might wish to engage in with their mentee. The payment offered to mentors for activities with their mentee was considered insufficient by a number of participants in the pilot, so it was increased for the RCT. This payment was intended to be contingent on mentors fulfilling all aspects of their role and PCs were asked not to give out the vouchers unless the mentors had actually met up with their mentee and completed their contact diary. However, PCs continued to pay mentors during the RCT, even if they had not seen their mentee, as they said they would have found it difficult to have withheld the money.

### Recruitment to the RCT

The modifications made to the recruitment procedures as a result of the pilot had some impact, but were not sufficient to achieve the target numbers (see above). Just over half of the intended number of mentees was recruited to the RCT (26 as opposed to the hoped for 48), and 14 mentors consented to the trial as opposed to the target of 24. LA3 had to withdraw from the study prior to commencement of the intervention as all their trained mentors dropped out, which meant that no mentoring relationships could be established.

**Figure 1:**
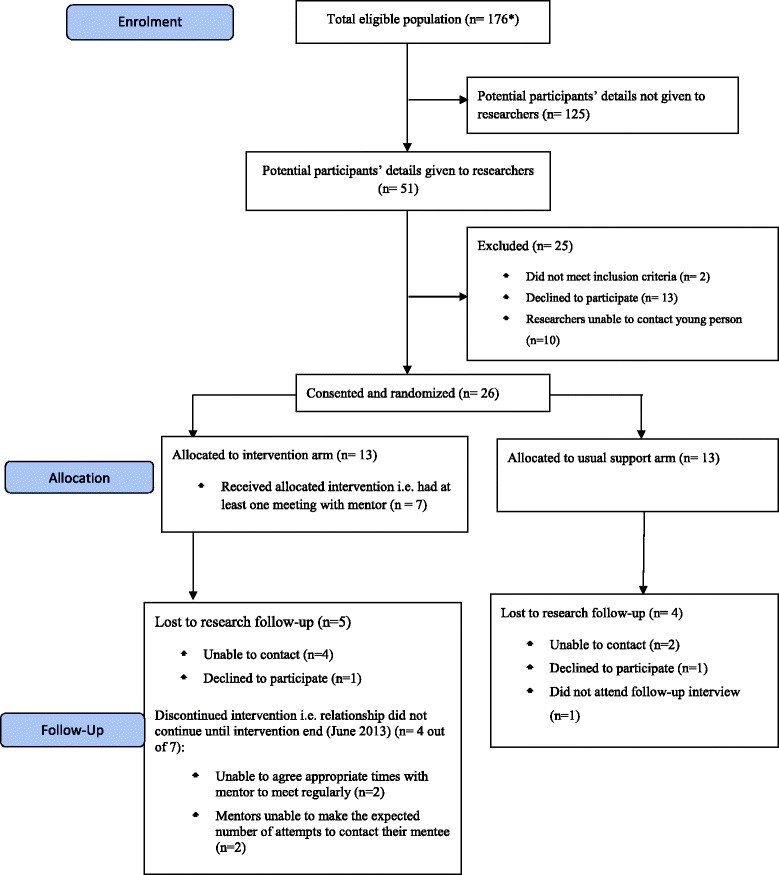
CONSORT flow diagram showing the flow potentially eligible participants (mentees aged 14 to 18 through the phase2 trial). *Based on number of female LAC aged 14-18 placed in borough across the three LAs as of March 2012.

**Figure 2:**
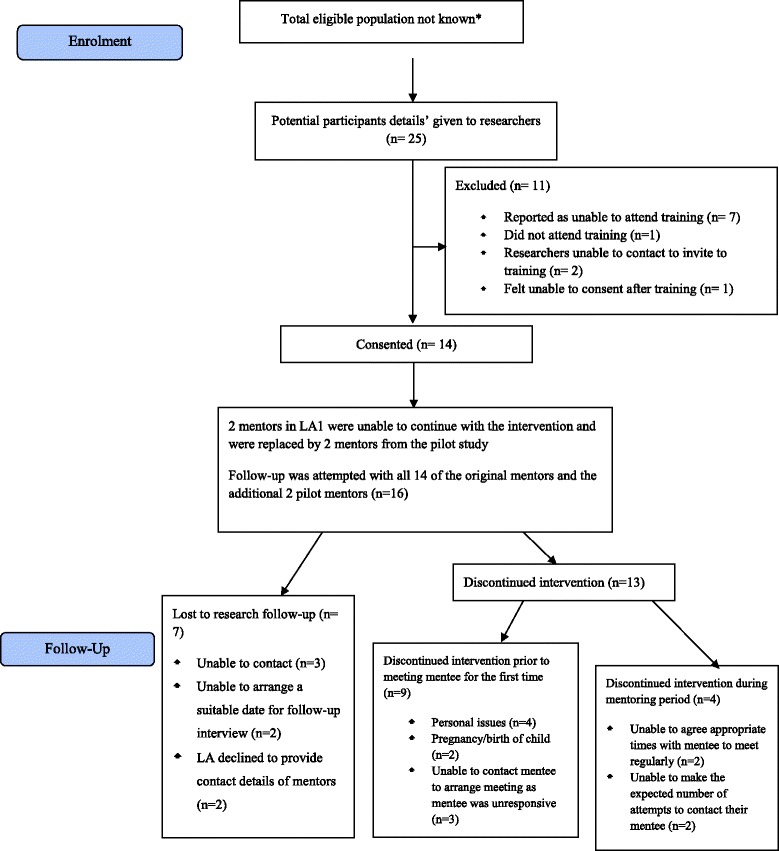
A CONSORT flow diagram showing flow of potential mentors through the phase 2 trial. *Social services do not have an available network of care leavers to recruit from

The flow of phase II participants, including retention rates to both the research and intervention, is illustrated in Figures 1 and 2. Further details of the intervention and study participants have been reported elsewhere [39].

### Lessons from the RCT

In this section we present the analyses of the qualitative data gathered from interviews at the end of the trial, together with the survey results (see Additional file 1).

#### Qualitative data analysis

Two researchers undertook an initial ‘open coding’ of a selection of transcripts from the interviews and focus groups. This involved assigning labels to data that indicated their relevance to the research questions being addressed. Following open coding, researchers adopted an iterative process [40] whereby they looked for patterns, similarities and differences in the coded data. This process was used to coalesce codes into seven descriptive categories that illustrated the range of barriers encountered in conducting this trial. The full set of interview, focus group and qualitative survey data was then organised using these categories. In a final stage of the analysis, undertaken through discussion among the authors, five analytical themes were identified that cut across those categories and offered an explanation of the underlying processes and dynamics [41] that challenged trial implementation. All analyses were conducted using the NVivo qualitative analysis software package [42]. The relationship between codes, categories and themes is shown in Table 1. It is the final set of explanatory themes that is presented in the results section below, illustrated with verbatim quotes from the data. Participant identifiers indicate the Local Authority (LA) from which the participant was recruited, the participant’s role (with an additional numeric identifier where necessary) and whether data were collected through an interview or focus group (G). Additional survey data are clearly labelled as such (see Table 1).

**Table 1:** **Table showing the development of analytical themes identified through interviews with mentees and mentors**

**Codes**	**Categories**	**Themes**
Recruitment and referral process: LA staff		1. Protective professionals: protecting vulnerable young women
Actual recruitment criteria used by LA		
Perspectives on the prescribed study recruitment criteria		
Understanding of the study recruitment criteria		
Recruitment material distribution	1. Variables interpretations of recruitment criteria	
Recruitment material format & content	2. Attitudes and perceptions of trials: LAC and care leavers	
Randomisation	3. Recruiting and retaining mentees	
Barriers to recruitment	4. Recruiting and retaining mentors	
Recruitment and referral process: young women	5. Acceptability of the intervention for LAC	2. Engaging and staying engaged (attachment, relationships and the mentoring intervention)
Methods of promoting the study to young women		
Engagement		
Changing nature of mentor-mentee relationship		
Matching		
Meaning and purpose of mentoring		
Mentor diary		
Mentees feelings towards mentor		
Mentoring intervention incentives		
Mentors feelings towards mentee		
Nature of mentor-mentee contacts		
Topics of discussion		
Views on structure & content of a		
mentoring programme for LAC		
Methods of promoting the study to LA staff	6. Attitudes and perceptions of trials: social care professionals	3. Protective professionals: prioritising more important work
Conducting or participating in non-CARMEN study research	7. Lack of LA research infrastructure	4. Cultural resistance
Participating in CARMEN study		
Research into teenage pregnancy among LAC		
Research outcomes		5. Structural deficits

#### Quantitative data analysis

Quantitative data from the online survey were analysed using descriptive statistics. All analyses were conducted using IBM SPSS Statistics v20 [43]. Survey analysis is presented below where it reflects themes generated through the qualitative analysis.

## Results

### Protective professionals: protecting vulnerable young women

The need to understand barriers to recruitment in the trial shaped both our additional data collection and our analysis. Social workers’ understandings and opinions about the purpose, process and need for randomisation in the study – in relation to the needs of the population they were supporting – offered some insight. Some social workers considered that randomisation, as applied to this group of young people, was reasonable ‘as long as they fully understood what they were making a decision about’ (LA3 social worker). However, others were unhappy about not being able to offer a potentially beneficial intervention to all young people and thought that the randomisation process potentially conflicted with their role as a service provider:

To me it’s blatantly obvious that having a mentor is going to be a positive beneficial effect, so it’s almost like saying, well how many people think that letterbox is red? Yes everybody’s going to nearly think it’s red because it’s blatantly obvious … so I actually think it’s an incredible waste of money and resources. (LA2 social worker interview)

A number of social care professionals considered it was unethical to raise expectations of vulnerable young people, which might then not be met, and expressed the view that any young person who showed any interest in the study should automatically receive the intervention. Concerns were also expressed that young people who had agreed to participate would feel let down if they were not allocated a peer mentor and this could affect their willingness to engage in services more generally in the future:

If they are going through this and then they feel that they are going to have a mentor, how do they respond to being told well you haven’t been allocated one after they’ve gone through that process. And sometimes it’s really hard for them to accept or say to themselves that you know, I do need a mentor and once they’ve made that step, how crushing is that then to say they haven’t got one? (LA2 health and education professionals focus group)

LAC responses to the survey suggested that randomisation, in itself, would not deter young people from participating in research. The majority of respondents (16 out of 21) stated they would be interested in taking part in this study, even if they only had a 50 per cent chance of receiving a mentor. Only one of the five respondents, who said they would not take part in a study of peer mentoring, cited randomisation as the reason.

The study participants had mixed understandings of randomisation. Data from the semi-structured interviews confirmed that although some of them recalled the purpose of randomisation, others could not remember what they had been told, or still had misconceptions about it, such as believing they had been allocated a mentor on the basis of their personal characteristics or behaviours, or based on resources, i.e. there were not enough mentors available or that the study was a ‘competition…to win a mentor’ (LA1 Mentee 1007 interview).

Of the ten young people interviewed directly after they were allocated to the usual support group, seven reported they were ‘ok’ with not having a mentor or ‘didn’t mind’; however, three said they were ‘disappointed’:

I was hoping that I did get one [a mentor]…I think I hoped too much though. So it was, I was fine about it afterwards, ‘cause I understood, but it was just like that fact I really wanted one. (LA2 Mentee 2002 interview)

Although clear criteria for the selection and recruitment of mentors and mentees were circulated to all participating LAs at the beginning of the study, it became apparent that many social workers were exercising their own judgment in determining who should be included, or excluded, from the process. In the professional focus groups social workers from the different LAs differed in the approach they took; LA1 social workers reported selecting only the most vulnerable, disengaged young women, who they considered to be ‘more at risk of becoming pregnant’ (LA1 senior manager), whilst LA2 social workers reported targeting their more stable and organised young people. Social workers in LA2 considered that mentoring would be particularly beneficial for the most troubled, isolated young people; however they were also most protective towards this group, in terms of being unwilling to expose them to potential harm or risk. Social workers in both Las acknowledged that it would be easier to go for young people who they knew would engage with services:

Two of the young people that you’ve been working with…those two were selected because they’re here [attending the local Education and Achievement centre]. Because they’re here, they come to everything, and you can engage them. And because they’re known to us, it’s almost like ‘right well we’ll use them then for this’ whereas actually there are a lot needier young people who are incredibly hard to reach and it’s them that need it. (LA1 health and education professionals focus group)

Again, our analysis suggested that a protective attitude to the population seemed to impact on recruitment to the study, in effect filtering or reducing the size of the population from which we were seeking to recruit.

### Engaging and staying engaged (attachment, relationships andthe mentoring intervention)

Our analysis of data from mentors, mentees, and other LAC and care leavers was indicative of a range of issues relating to engaging with both the study and the intervention and then remaining engaged for the duration of the project. The majority of mentees and mentors reported having experienced problems in relation to disrupted attachments, loss, rejection or abuse prior to going into care. Qualitative analysis suggested that any difficulties participants might experience, in developing trusting, committed and consistent relationships in their wider lives, might similarly apply to forming relationships with adults in positions of authority, including with the researchers (in relation to recruitment and retention to the study) and with each other (in the context of the peer-mentoring relationship).

Once identified as potential participants, setting up consent meetings with young people proved extremely challenging. Numerous reminders were usually needed before a date and place could be agreed upon and even then we had to rely on social workers or foster carers to ensure the young person attended these meetings. In many instances the young person (mentee) would fail to turn up and meetings had to be rearranged. This happened even when the young person had expressed enthusiasm about participating.

Our experiences were consistent with those of many of the LA professionalstrying to engage this group of young people in services. With regard to this study specifically, some social workers wondered whether the difficulties in recruitment and retention might relate to the negative connotations of being singled out for an intervention, where the primary stated aim was to reduce teenage pregnancy. Others suggested that young people probably viewed this as ‘just another process that they have to go through that makes them feel different’ (LA3 social worker) and considered that non-attendance might be a way of registering some form of protest:

I think by that stage some young people are really suffering from professional burnout, so, they just don’t want to…‘I don’t want to hear…don’t tell me about another person that you’re going to refer me to. I’ve already got a youth offending officer, a probation officer, a social worker, a keyworker, I’ve got somebody from children looked after health…my teacher, my school nurse’. You start potentially going into double figures. (LA2 social worker interview)

One PC expressed the view that LAC may find it hard to vocalise their opinions about whether or not they want to participate because their experiences leave them feeling disempowered; they therefore end up voting with their feet, by not turning up, or not responding to phone calls, rather than communicating in a more straightforward way:

We need to probe with young people about what they really want. I think some people – particularly children in care, and that goes for mentors as well as mentees…it’s the sense about they’re not really empowered to say actually ‘I don’t want to do that, I’d like to do this’ or they’re offered things and … they feel as if they can’t say *no*. (LA2 PC1 interview)

Professionals reported that it can take a long time for LAC to build positive relationships and this view was reinforced by some young people, who expressed a suspicion that the research was just yet another service they were being forced to engage with by social services:

I thought it was gonna be like time-wasted and like, like how the social workers do it; like ask a load of things – I just thought it was the random things that the social workers just have to do. (LA1 Mentee 1003 interview)

It takes me a while to get close to someone and become friends with someone, or until I trust someone. I thought it’d be hard for me to do that. (LA1 Mentee 1001 interview)

Retention of LAC mentees to the intervention was similarly problematic. Perhaps not surprisingly, given their early life experiences, LAC may find it particularly hard to establish routines or to structure their lives. Mentees would often agree to attend a meeting with their mentor, but would then alter the time or place, without notice, or simply fail to turn up and appeared to lack the motivation or willingness to turn up:

‘Cos sometimes like I’ll have one of them lazy days when I’ll just…don’t want to go nowhere and I just want to stay in my house and…It’s like if she came to get me – I know it sounds lazy, but if she came to get me then obviously I wouldn’t mind going, but I don’t really…I like travelling but sometimes I don’t. (LA1 Mentee 3 interview)

Focussing the intervention on a reduction in teenage pregnancy rates may also have distanced young women from the study. A number of mentees were not interested in talking about relationships, sex or contraception at all, either because they did not have a boyfriend, or because they thought their education, or other issues, were more important:

‘Cos I think of school and education first and studying; that’s the…like the last thing on my mind [sex and relationships]. (LA2 Mentee 2002 interview)

I spoke to her about me moving and everything and told her I felt really lonely and everything, ‘cos obviously I’m living in a flat on my own. (LA1 Mentee 1006 interview)

Our analysis suggested that a similar range of issues had impacted on mentor recruitment and retention, as well as the fact that, as most LAC disengage from the care available to them at 18 years of age (unless in full time education), they were less easy to access compared with their younger counterparts:

I don’t know whether or not that’s just about young people moving on and disengaging with social services ‘cos they don’t want the stigma of us being involved in their lives you know, some young people simply move on and don’t stay in touch with anyone…perhaps, they feel they’ve had so much kind of social services involvement that that’s sort of enough for them. (LA1 PC1 interview)

Thirty out of 34 (88%) care leavers who responded to our survey said they would be interested in becoming a mentor for young people in care. However, in practice, the care leavers recruited to the trial found it hard to fit the mentoring in around their existing commitments; indeed some had decided against participation because they were concerned about their ability to commit to the training or to do justice to the mentoring role. Despite their best intentions, personal and work-related issues repeatedly impacted on mentors’ ability to fulfil commitments.

### Protective professionals: prioritising more important work

Our analysis suggested that social work professionals were not just protective of the young women they supported; they were also protective of their time as professionals, particularly when research demands threatened to impinge on the generic social work duties. Prior to commencing the pilot and the exploratory trial, the research team had held a number of meetings with senior managers from the participating LAs to ensure they understood what was required and to establish their wish and ability to deliver the intervention. We were clear that active support from senior management would be crucial for the effective delivery of the project. All of the senior managers in the participating LAs felt they would be able to deliver what was being asked, including freeing up sufficient time for a PC to spend on the project – estimated at being around half a day each week.

As the trial progressed, however, it became clear that social workers were finding it hard to deliver the study as intended, because of the competing demands of their generic work commitments, as well as other organisational pressures and disruptions, such as frequent re-structuring and regular inspections:

It’s about trying to continue to get social workers to prioritise it. Because they are constantly getting other needs, other issues, child protection…I think the general concept has been received well and most people think it’s a good idea but it’s then the effort it needs to actually translate that into something active and meaningful. (LA2 Senior Manager interview)

I think time was probably the biggest obstacle because it was a competing task with, you know, the statutory duties that we have. So often when I went back to people it was oh yes I need to speak to so and so but I haven’t yet because I’ve been busy doing this…people could see the value of it but it was not top of the priority. (LA3 PC1 interview)

Recruiting LAC to the study was perceived as an additional and unwelcome burden, particularly for the PCs:

Despite sending out a number of emails to both teams, I have not heard back from any social workers as yet and the deadline I gave them was last week. I am still working on it; however my other work has taken precedence this week. (Email communication from LA1 PC1)

### Cultural resistance

At a senior management level, there seemed to be an appreciation of the value of the research for both the LAs and the young women they supported. Although senior managers acknowledged that it might be difficult to engage young women in the study and expressed some concerns about their vulnerability, they also saw the potential benefits of the study, in terms of providing young people with better opportunities, sexual health knowledge and choices in their lives. However, social workers on the ground, appeared to view the research as an additional burdensome imposition on their already pressurised time. Social work professionals often found it difficult to understand the need for RCTs in developing an evidence base for practice, the concept of equipoise was generally not understood, and there were variable interpretations of the rationale for randomisation. For example one social worker (LA1) thought that the reason for randomisation was a ‘resource issue’ (i.e. a lack of mentors available for all participants), while another thought we were measuring ‘how effective the computer matches [are] in comparison to the matches that you make yourself’ (LA1 social worker). For one social worker, feelings about randomisation extended to a more generalised mistrust of research and academics:

And then usually what’ll happen after all that wasted money and resource [spent on academic research], they’ll bring out some paper or form for us to do something else, which’ll just clog up 85, 95 per cent of our work doing something that’s not necessary. So I find it…it’s just a waste of time, waste of money. I understand the need for a little bit of research, because that’s how things come out, but I think we’ve gone research bureaucracy mad at the moment. (LA2 social worker interview)

Such views can undoubtedly be found in other professions and are not unique to social workers. However, it is still the case that the majority of social care policy makers and social work academics still regard RCTs as ethically, methodologically and logistically inappropriate [8,44]. It is perhaps not surprising therefore that few social workers qualify with an understanding of this methodology, and many qualify with only a limited appreciation of the importance of *doing research* in order to generate the evidence base that most appreciate is important. Commissioning RCTs in agencies where there is clearly a need for a ‘hearts and minds’ initiative, if not considerable education about the nature of controlled studies and what is required of their implementation, is hampered by the very real day-to-day pressures faced by staff and a historic lack of investment in building and supporting the organisational capacity required to conduct high quality research. We turn next to this issue.

### Structural deficits

As the study progressed and it became increasingly clear that PCs were struggling to meet the recruitment targets, the researchers had to become more actively involved in the identification and recruitment of mentors and mentees than was specified in the original protocol. Researchers attended social work team meetings in the three participating Las, to brief social workers on the study, allay any concerns and encourage referrals. The research team also sought to enhance recruitment by publicising the study more widely to other health and education professionals working with LAC within the LAs, asking them to forward names of potential candidates. In addition, the research team regularly contacted senior managers to ask them to prompt social workers to identify potential participants. One of the PCs felt that all responsibility for managing the task locally had been delegated down to her. She had expressed frustration about the fact that, without active senior management backing, her requests to social workers to put forward names of LAC, who might be suitable for the study, went unheeded:

And it didn’t filter down. With [LA name], it needs to come from the top, going down, to get responses. And because I’m down [laughs] and I’m trying to…get up, getting everybody to participate in this, it wasn’t working. So the minute that [name] got involved, yeah, things started to move a lot faster. (LA1 PC1 interview)

During the intervention, PCs said they had found it difficult to commit sufficient resources or emotional support to the mentors and considered that, in order to deliver the intervention effectively, a dedicated project manager would be required. Although mentors reported receiving helpful advice from PCs when they were able to contact them, in LA1 they said the PC was difficult to contact and too busy to support them:

To be honest I could have had more support…but whenever I did manage to get hold of her, ‘cos she’s a very difficult person to get hold of, when I did manage to get hold of her…. she will give advice, I’ll give her tops for that. But I still think I could have had a bit more support. (LA1 Mentor 4 interview)

It became clear as the study progressed that PCs were simply unable to deliver on the key aspects of the intervention without dedicated time and resources being allocated to free them up from other aspects of their generic work. The existence of a Research and Development research infrastructure might have helped address some of the structural barriers identified.

## Discussion

### Summary of main findings

We have presented findings from an analysis of qualitative data derived from interviews and focus groups with social workers and LAC participating in an RCT, as well as data derived from a survey of LAC and care leavers, which illustrate some of the problems associated with conducting a trial involving LAC. Many of the problems we encountered are similar to those described in other studies of hard-to-reach and hard-to-engage populations [7,12]. Only 54 per cent of the target recruitment of LAC was achieved and retention to the intervention was low, with only three mentor-mentee pairs completing the 1year intervention in the trial. LAs lacked the infrastructure, or resources, to be able to deliver the trial effectively; there was variable understanding and acceptance of the research methodology and inclusion criteria and varying ability, or perception, of the need to prioritise the research against other work.

We found no evidence that randomisation in itself deterred young people from participating in research, nor were they concerned about possible harm as a result of not being allocated a mentor (usual support arm). Having said that, several ‘usual care’ participants expressed disappointment at not receiving the intervention, a finding that has also been noted by studies in health, education and the social sciences [45-47]. Furthermore, the notion of randomisation appeared to be one that many young people, even those who participated in the trial, found hard to understand. Our findings are consistent with the difficulties in understanding concepts of equipoise and randomisation in the general public [48] and the recruitment challenges in trials of social interventions. Particular care needs to be given in future RCTs to ensuring that young people fully understand the rationale for randomisation when obtaining consent.

It has been suggested that provision of clearer written information to all participants and allocating sufficient time to discuss the trial in depth may help to address some of the widespread misconceptions about the nature of trials and randomisation [49]. In this study information leaflets for the young people were scrutinised and in some cases substantially re-written by our service user (former LAC) who was a member of our advisory group, to try and ensure that the format and content were attractive, intelligible, clear and simple, yet sufficiently detailed to ensure all necessary information was made available. However, RCTs of complex interventions are, almost by definition, somewhat difficult to distil down to an essential clear essence. This can be a particular challenge when researching certain groups, for example young people or the very elderly and individuals with severe mental health problems, who may require more time and attention paid to ensuring communication is accurate, appropriately expressed and fully understood.

Whilst the prospect of randomisation did not appear to deter young people in care from participating in the trial, many social workers expressed concerns about its use in this population. Senior managers in social care were generally more supportive of the need for high-quality research than practitioners; however this theoretical acceptance of the need for randomisation and for conducting trials in social care settings was in stark contrast to what happened in practice [15]. Social workers were challenged by the practical problem of identifying time to allocate to the demands of the study, as well as what many of them considered to be the harmful and even unethical aspects of randomisation with such a vulnerable population. There was some evidence that these considerations had deterred some social workers from approaching or referring eligible young people, even though the research team actively tried to raise awareness and provide information about the reasons for randomisation.

It has also been noted that, because both health and social care interventions require an element of organisational restructuring to implement, they can engender a degree of cultural resistance [7]. As has been noted in previous studies, social workers acted as informal gatekeepers [5,7,8]and tended to select certain individuals based on a perception of who may or may not be capable of participating in the research or who would benefit most (or be least harmed). This approach is not only likely to introduce selection bias, but also denies children and young people the opportunity to participate in research and to have access to new interventions that may be efficacious. Denying children this opportunity opposes the growing consensus that children’s views should be taken into account on matters that affect them [4,50], although clearly there is a balance to be struck between conducting research and protecting this highly vulnerable population.

Previous literature on conducting trials with hard-to-reach populations has focussed on difficulties in recruitment [7,12]; however it is clear that it is not enough to just be able to access LAC and care leavers, but that they continue to require a lot of support to sustain their engagement throughout the intervention. Overall the LAs were unable to provide the required amount of support to the care leaver mentors during the intervention; had this been in place then it is possible that retention rates to the intervention may have been higher.

### Implications of the study

The difficulties encountered in implementing this trial raise questions about the transferability of trial methodology to settings outside of health, most notably in social care, but also in education and some areas of public health. Even though randomised trials in education, crime and social work were in existence, before their introduction in medicine [51], the largely negative results of these early trials tended to be attributed to the use of an unsuitable methodology, rather than an ineffective intervention; see also [52-54]. Very similar barriers to recruitment to those we describe, have been reported in trials conducted in health care settings, including misconceptions about trials; lack of equipoise; misunderstanding of the trial arms; variable interpretation of eligibility criteria; time constraints; lack of reward and recognition and paternalism in an RCT looking at employment difficulties in individuals with severe mental illness [12,16,49,55].

The impact of professionals’ concerns about patients and belief in the effectiveness of the interventions has resulted in the subversion of randomisation in trials of health interventions [56].

After describing three randomised trials of social interventions conducted in London, England, Oakley and colleagues [5] suggest the following strategies to conductsuccessful trials of social interventions: focussing on priority issues for trial participants; having a clear scientific and policy rationale for using random assignment; allowing sufficient time for detailed discussions with those who need to ‘sign up’ for randomisation, combined with sensitivity to the perspective of key stakeholders; careful piloting of recruitment and informed consent procedures that explain the design in accessible ways; considering the position of control groups and adequate resourcing of development time.

Our findings also identify a distinct resourcing issue not routinely encountered in RCTs in health, which has implications for recruiting LAC, and possibly other groups, to future trials in social care contexts. NHS Trusts support the cost of providing an experimental intervention, and health care commissioners have a commitment to provide funding to support NIHR-funded research. A research infrastructure is also in place that provides research support funding to cover the cost of NHS Trust staff time spent recruiting participants. There is no equivalent provision in social care. The agreement of senior management to participate in the study is not sufficient to guarantee the necessary resources (such as time and funding for a project manager) to secure its implementation. Had this trial taken place in a healthcare setting, the researchers could have included within their budget the funding for a dedicated healthcare professional to recruit participants as a research support cost, enabling the host Trust to backfill that time. The absence of such provision in social care meant that PCs and social workers were constantly having to juggle the competing demands of the research and their generic duties, which in this study took priority. Recruitment and retention rates in any future Local Authority-based trial, will not improve until they receive research costs in the same way as currently provided for NHS trials. This would enable them to employ the equivalent of a research nurse to take responsibility for identifying, enrolling and retaining participants without competing demands on their time. Additional input and support from senior managers to encourage a sense of ownership of any trial are important. However, as has been noted in other studies, approval from senior managers (the official gatekeepers) does not necessarily guarantee the cooperation or commitment of the informal gatekeepers and participants in the study [4,7]. The engagement of social workers at an early stage of discussions about any proposed research and in the research process is also likely to prove beneficial [57].

Our findings also have implications for engaging LAC and care leavers in trials, in terms of the purpose of the intervention and the value placed on the stated outcomes. Although high rates of teenage pregnancy in LAC were, at the time of developing this programme, a priority issue for Government and arguably for health and social care providers, it was not viewed as such by the young people themselves. There is a need to ensure that the voice of young people is sought and heard more loudly when designing and delivering interventions such as this. The peer-mentoring intervention itself was informed and shaped by a targetted review of the literature around Looked After Children and Interventions to reduce Teenage Pregnancy. Moreover there was evidence that a peer-mentoring approach had been effective and acceptable in other populations, including in relation to smoking prevention in adolescents [58] and in sex education [59]. The applicants consulted extensively with agencies, individuals and organisations working with LAC including: The Who Cares Trust and the NCB. We also held a series of meetings with the Heads of Children’s services and senior social workers in the three participating Local Authorities, which were used to refine and, in some cases, re-design aspects of the intervention. Further, one of the members of the Advisory Group, which met six times during the course of the 30-month project, had been recruited as a ‘service user’ and was able to use her experience of the care system to contribute to ensuring that the aims, methodology and interpretation of results reflected and took into account the views of young people in the care system. Feedback from the young people who were participating in the intervention was collected through the mentors’ monthly support meetings and also at the end of the intervention, by individual- and group-based discussions with mentees, young people from the care as usual arm and also mentors. Further feedback was obtained from young people through the National Surveys into LAC and care leavers’ views of peer mentoring and of the randomisation. Arguably, however, the views of these young people were sought too late to make a significant contribution to the development or design of the peer-mentoring intervention and should be instituted at an earlier stage in the future.

As Oakley and colleagues [5] suggest, the personal significance attributed to an issue by participants is particularly important in social interventions. This highlights the importance of a shared common goal and sense of purpose: a hard-to-do trial, with a hard-to-reach population was arguably made even harder by having the stated outcome that of reducing teenage pregnancy. It is possible that if a more tangible, important or relevant goal had been offered to participants, e.g. supporting their transition out of care into independence or educational/vocational support, the take-up rate would have been higher. The research agenda needs to be more informed and driven by the stated needs of young people themselves, rather than by external agencies.

## Conclusions

There have been calls in recent times for more trial methodology to inform service delivery within social care – including at a policy level, with the setting up of the National Institute for Health and Social Care Excellence Collaborating Centre for Social Care Guidance. Whilst eminently desirable, the problems that we and others have encountered in trying to mount RCTs within social care settings suggest that these aspirations may be difficult to achieve in reality.

Many of the challenges in mounting an intervention in a Local Authority have been described in other areas, such as education and health; however this article also highlights the lack of a research infrastructure in social care, which is widely recognised as essential for the successful implementation of high-quality research in health, including, though not confined to, randomised trials. We have also tried to highlight the cultural challenges within social work, as evidenced by ambivalence and sometimes antipathy towards research, and a limited awareness, understanding and acceptance of trial methodology. In 2009, in light of the growing interest in using RCTs to evaluate social interventions in children’s lives, Hobbs et al. [60] emphasised the importance of building capacity for conducting RCTs and addressing the concerns of the practitioners who often act as gatekeepers to vulnerable and hard-to-reach clients. We endorse these recommendations.

Whilst many of the same problems continue to be encountered within health care research [61], the ‘distance to be travelled’ within social care remains considerably further.

## Additional file

Additional file 1:
**Young People’s National Online Survey 14-18.**

## Abbreviations

DCS: Director of children’s services
EBP: Evidence-based practice
LA: Local authority
LAC: Looked after child/children
NIHR: National Institute for Health Research
PC: Project coordinator
RCT: Randomised controlled trial
