# Supplementary material for: Challenges to undertaking randomised trials with looked after children in social care settings
Source: Trials. 2015 May 7;16:206. doi: 10.1186/s13063-015-0708-z (PMC4486703; doi:10.1186/s13063-015-0708-z)
Supplement: Additional file 1: — Young People’s National Online Survey 14-18. [file 13063_2015_708_MOESM1_ESM.doc]

**Additional file 1**

Young people’s national online survey 14-18

Are you:

Aged 14 – 18

Female

In care

Live in UK

If no –

Participants eligible to take part in the Carmen Study are young women aged 14- 18 who are currently in care and young women aged 19-25 who have been through the care system. To be eligible to complete the survey you must also fall into these categories. Unfortunately you do not. Thank you for your interest in the Carmen Study.

If you would like to know more about the study for your information, please email [carmen@sgul.ac.uk](mailto:carmen@sgul.ac.uk)

BLURB:

St George’s University of London, together with other partners, are being funded by the Government to do a Research Study. We are trying to find out whether meeting ‘peer mentors’ can have a positive impact on the lives of young women aged 14-18 in care. These positive effects could be to their general wellbeing, social life, relationships, attitudes to sex and thoughts about early pregnancy.

In this research study, a peer mentor is a young woman aged 19-25 who has been through the care system herself.

By completing this survey you will help us to understand what young women in care think and feel about the research methods we are using and peer mentoring. The Carmen Research study is currently being delivered in 3 local authorities and is not recruiting further participants at present, but we hope to be able set up another similar study in the future.

We really want to hear your views as to whether peer mentoring for young people in the care system is practical and useful. If it is, the research team will recommend that peer mentoring for young women in care becomes more widely available.

This survey should take you about 15 minutes to complete. There are 8 sections. Your responses will be stored confidentially. In reporting of the survey findings we may report something that you have said but no one will be able to identify it was you who said it.

If you complete every question of the survey you can enter into a prize draw to win a £30 Love2Shop voucher which you can spend in a variety of shops.

To do this, you will need to give us your first name and an email address (or a telephone number). This information will be stored separately from your responses so that your answers remain anonymous.

Thank you for completing this survey.

The Carmen Research Team.

SECTION 1:

Age …….

How would you describe your ethnicity? (please tick one)

- White or White British
- Mixed ethnicity
- Asian or Asian British
- Black or Black British
- Chinese
- Not sure
- Other please state ………..

What part of the UK do you live in?

- North England (N. East and N. West)
- Yorkshire and the Humber
- Midlands (East and West)
- London
- South England (S. East and S. West)
- East of England
- Wales
- Scotland
- Northern Ireland

How many times have you been placed in care?

N = …….

If once, how long have you been in care?

Years ….. Months ……. Weeks ……..

If more than once, what is the longest period of time you have spent in care?

Years ….. Months ……. Weeks ……..

SECTION 2:

Do you have or have you ever had a mentor?

Y/N

(N:B Sarah – we could have some sort of ‘help’ or ‘i ‘ button here for definition. Oxford Advanced Learners Dictionary online says a mentor is ‘an experienced person who advises and helps somebody with less experience over a period of time’. <http://oald8.oxfordlearnersdictionaries.com/dictionary/mentor>

IF YES CONTINUE SECTION, IF NO SKIP TO SECTION 3

If you have more than one mentor, please answer in relation to the mentor that you have most contact with.

Where is/was your mentor from? My mentor is someone from ……(Please tick one)

- School / college / other education centre
- Youth centre / Connexions
- Mental Health Service
- Advocacy Service
- Religious organisation
- Business organisation
- Youth Offending Team / Youth Justice Service
- In care / a care leaver
- Other (please specify) ………………………….

Is/was your mentoring provided in a group setting or on a one-to-one basis?

(please tick one)

- Group
- One-to-one
- Both

How often do/did you meet with your mentor? (please tick one)

- More than once a week
- Once a week
- Every two weeks
- Once a month
- Every two months
- Less than every two months

What do/ did you do during your time with your mentor?

(Tick all responses that apply)

- Sit and talk with them
- Home work / Education
- Leisure activity e.g. eating a meal out / cinema / sport etc
- Attend an appointment e.g. Doctor / Social Worker / Clinic
- Other (please specify)……………………………………….

How long will you / did you have a mentor for?

- Less than 6 months
- 6 months to 1 year
- 1 year to 1.5 years
- 1.5 years to 2 years
- More than 2 years

Do/did you enjoy having a mentor? Y/N

Please explain what you like/d about having a mentor

……………………………………………………………………………………………………………………………………………………………………………………………………

Please explain what you do/did not like about having a mentor

……………………………………………………………………………………………………………………………………………………………………………………………………

If your mentoring has ended, how did you feel when it finished?

……………………………………………………………………………………………………………………………………………………………………………………………………

SECTION 3:

The Carmen Study aims to provide young women in care with a supportive relationship with their mentor and help them make informed choices.

If you had an opportunity to choose a mentor, what would they be like?

For each question a – f below, please choose one option and then rate how important each one is to you.

a) I would like my mentor to be:

- Male
- Female
- I don’t mind whether my mentor is male or female

Now rate how important the gender of your mentor would be to you

(Please circle a number)

| Not at all important Somewhat important Very important |
| --- |
| 1 2 3 4 5 |

b) I would like my mentor to be:

- Age 14-18
- Age 19-25
- 26 – 30
- 31 – 40
- 41+
- I don’t mind what the age of my mentor is

Now rate how important the age of the mentor would be to you

(Please circle a number)

| Not at all important Somewhat important Very important |
| --- |
| 1 2 3 4 5 |

c) I would like my mentor to be

- Someone who is in care or has left care
- Someone who has a profession working with children in care e.g. participation officer / advocate
- Someone who has no experience of the care system
- I don’t mind what experiences the mentor has had

Now rate how important the mentor’s experience of the care system would be to you

(Please circle a number)

| Not at all important Somewhat important Very important |
| --- |
| 1 2 3 4 5 |

d) I would like my mentor to

- Have the same religion as me
- Have a different religion to me
- I don’t mind what their religion is

Now rate how important the mentor’s religion would be to you

(Please circle a number)

| Not at all important Somewhat important Very important |
| --- |
| 1 2 3 4 5 |

e) I would like my mentor to

- Have the same ethnicity/culture to me e.g. shared country/region of origin
- Have a different ethnicity/culture to me
- I don’t mind what their ethnicity/culture is

Now rate how important the mentor’s ethnicity/culture would be to you

(please circle a number)

| Not at all important Somewhat important Very important |
| --- |
| 1 2 3 4 5 |

f) I would like my mentor to have

- The same interests as me e.g. music / films
- Different interests to me
- I don’t mind what their interests are

Now rate how important the mentor’s interests would be to you

(please circle a number)

| Not at all important Somewhat important Very important |
| --- |
| 1 2 3 4 5 |

g) Please tell us if there is something else that would be important to you in a mentor

……………………………………………………………………………………………………………………………………………………………………………………………………

If you were offered a female mentor aged between 19 and 25 who has left care, would you want one?

Yes / No

If yes, please explain what your reasons would be for wanting the mentor.

……………………………………………………………………………………………………………………………………………………………………………………………………

If no, please explain what your reasons would be for not wanting the mentor.

……………………………………………………………………………………………………………………………………………………………………………………………………

SECTION 4:

The Carmen Study is a research study. Young women who want to take part in the research participate in a confidential interview with a researcher. The researchers have to ask quite personal questions, for example about their care history, relationships and attitudes towards sex and pregnancy. The reason for this is to find out about the experiences and views of the participants who take part in the study.

How comfortable would you feel about answering questions of this nature in a research interview?

| Not at all comfortable Somewhat comfortable Very comfortable |
| --- |
| 1 2 3 4 5 |

Please tell us if there is anything that could be done to encourage you to take part in a research interview like this?

(SARAH PLEASE MAKE SURE THIS QUESTION IS ON SAME PAGE AS ABOVE)……………………………………………………………………………….

………………………………………………………………………………

SECTION 5:

After the research interview, young women have a 50% chance of receiving a mentor. Whether or not they receive a mentor is decided at random by a computer. The decision has nothing to do with any personal characteristics or behaviours of the young woman. The reason we are doing the research in this way is because we need to compare the experiences of those who receive a mentor with those who do not.

If the Carmen Study was available in your area, and knowing that you would have a 50% chance of receiving a mentor, would you still take part in the research study?

Y/N

If yes, please explain what your reasons would be for taking part in the research study.

…………………………………………………………………………………………………………………………………………………………………………………………………… If no, please explain what your reasons would be for not taking part in the research study.

……………………………………………………………………………………………………………………………………………………………………………………………………

How would you feel if you took part in the research interview but you didn’t receive a mentor?

……………………………………………………………………………………………………………………………………………………………………………………………………

SECTION 6

Young women in the Carmen Study are asked to meet with their mentor once a week, in person, for one year.

Do you think once a week is: (please circle / tick one)

- Too little
- Just right
- Too much

Do you think you would have any difficulties in meeting up with a mentor once a week?

Y/N

IF YES, what are those difficulties? (Please circle / tick one option)

- Education / work commitments e.g. homework
- Organised activities e.g. sports club / music lesson
- Other commitments e.g. time for friends

If you have ticked other: Please tell us what …………………………...

THIS QUESTION WILL BE SEEN BY 14/15 YEAR OLDS ONLY

Does you carer allow you to travel alone on public transport?

Y/N

THIS QUESTION WILL BE SEEN BY EVERYONE

Are you comfortable travelling alone on public transport? (e.g. bus / train)

Y/N

If yes, how far would you be willing to travel on public transport to see your mentor once a week?

- Nowhere – my mentor should come to me
- Less than 30 minutes
- 30 mins
- 1hr
- 1.5 hrs
- 2 hours
- As long as it takes as it doesn’t matter how far away they live

SECTION 7

Now imagine you are taking part in the Carmen Research Study and you have a mentor

Your mentor is a young woman aged between 19 and 25 and has been through the care system herself…..

How often would you like to meet up with your mentor?

- Once a week
- Every two weeks
- Every three weeks
- Once a month
- Less than once a month

What kind of contact would you prefer to have with your mentor in between face to face meetings with them? (check at most 2 answers)

- Telephone conversation
- Email
- Text messaging
- Other (please state) …………………………..

How long would you like to be in contact with your mentor? (pick one)

- Less than 6 months
- 6 months to 1 year
- 1 year to 1.5 years
- 1.5 years to 2 years
- More than 2 years

During meetings with your mentor, what would you like to do?

……………………………………………………………………………………………………………………………………………………………………………………………………

Once you had got to know your mentor, do you think you would be willing to discuss personal or emotional problems with them?

Y/N

Once you had got to know your mentor, do you think you would be willing to discuss sex and relationships with them?

Y/N

The relationship with your mentor has been ongoing for 11 months and it is going to end in a few weeks. Is there anything special that ought to happen when the relationship finishes?

Y/N

Would any of the following be helpful?

(Choose up to 2 of the following answers)

- Ensure there is a plan in place so I have other support when the relationship ends
- Ending ceremony with certificate
- A gift / letter
- A special activity of my choice
- Anything else please state ………………………………………………..

SECTION 8

Imagine you see a poster about the Carmen Study with details about the study and the contact details for the project coordinator, whose role is to recruit young women to the study.

You are interested in taking part. Would you feel able to call the project coordinator?

Y/N/ not sure?

How would you prefer to be first contacted with information about the Carmen study?

- In person
- Phone
- Email
- Post
- Social network site e.g. facebook or twitter
- Other (please state) ………………………

Who would you prefer to first contact you about the Carmen study? (choose one)

- My social worker or a previous social worker
- Another professional that I have contact with e.g. teacher / personal advisor
- A researcher
- The Carmen study project coordinator (even if I don’t know them)
- A Carmen Study mentor
- My carer / family member
- It don’t mind who I receive initial information from
- Other (please state)

We would like some more ideas about how to reach young women in care to tell them about the Carmen Study.

Please tell us about places young women in care might visit where the project coordinator or researchers could let them know about the study or put up posters for them to see.

………………………………………………………………………………………….

………………………………………………………………………………………….

BLURB:

Thank you for completing the Carmen Research Study Questionnaire.

By completing this survey you have helped us to understand a bit more about what young women in care think and feel about the research and peer mentoring.

If you would like to be entered into the Prize Draw to win £30 in Love to Shop Vouchers, please complete your name and contact details below. The winner will be randomly drawn from completed questionnaire entries on or soon after the 14^th^ December 2012 and will be contacted straight away.

Name

Email (Essential so we can email to let you know if you have won)

Phone (not essential)

If you have any questions, you can contact us on [carmen@sgul.ac.uk](mailto:carmen@sgul.ac.uk)

Or:

Deborah Meyer, Research Trial Manager on 02087255523

Fiona Clare, Research Assistant on 02087252625

Young people’s national online survey 19-25

Are you:

Aged 19 – 25

Female

Been in the care system

Live in UK

If no – END

BLURB:

St George’s University of London, together with other partners, are being funded by the Government to do a Research Study. We are trying to find out if providing peer mentors to young women aged 14-18 in care can have positive effects - in relation to their general wellbeing, social life, relationships, attitudes to sex and thoughts about early pregnancy.

In this research study, a peer mentor is a young woman aged 19-25 who has been through the care system herself. The peer mentors in the Carmen Study are young women aged 19-25, like you, who have been through the care system.

By completing this survey you will help us to understand what young women in care think and feel about the research methods we are using and peer mentoring. The Carmen Research study is currently being delivered in 3 local authorities and is not recruiting further participants at present, but we hope to be able set up another similar study in the future.

We really want to hear your views as to whether peer mentoring for young people in the care system is practical and useful. If it is, the research team will recommend that peer mentoring for young women in care becomes more widely available.

This survey should take you about X minutes to complete. There are 9 sections. Your responses will be stored confidentially. In reporting of the survey findings we may report something that you have said but no one will be able to identify it was you who said it.

If you complete every question of the survey you can enter into a prize draw to win a £30 Love2Shop voucher which you can spend in a variety of shops.

To do this, you will need to give us your first name and an email address (or a telephone number). This information will be stored separately from your responses so that your answers remain anonymous.

Thank you for completing this survey.

The Carmen Research Team.

SECTION 1 :

Age …….

How would you describe you ethnicity? (please tick one)

- White or White British
- Mixed ethnicity
- Asian or Asian British
- Black or Black British
- Chinese
- Not sure
- Other please state …………………………

What part of the UK do you live in?

- North England (N. East and N. West)
- Yorkshire and the Humber
- Midlands (East and West)
- London
- South England (S. East and S. West)
- East of England
- Wales
- Scotland
- Northern Ireland

SECTION 2:

Do/have you ever had a mentor? Y/N

(N:B Sarah – we could have some sort of ‘help’ or ‘i ‘ button here for definition. Oxford Advanced Learners Dictionary online is ‘an experienced person who advises and helps somebody with less experience over a period of time’. <http://oald8.oxfordlearnersdictionaries.com/dictionary/mentor>

IF YES CONTINUE SECTION, IF NO SKIP TO Q HAVE YOU EVEN BEEN A MENTOR FOR SOMEONE ELSE?

If you have/have had more than one mentor, please answer in relation to the mentor that you have/had most contact with.

Where is/was your mentor from? My mentor is/was someone from ……..

(please tick one)

- School / college / other education centre
- Youth centre / Connexions
- Mental Health Service
- Advocacy Service
- Religious organisation
- Business organisation
- Youth Offending Team / Youth Justice Service
- In care / a care leaver
- Other (please specify) ………………………………………..

Is / was your mentoring provided in a group setting or on a one-to-one basis?

- Group
- One-to-one
- Both

How often do / did you meet with your mentor?

- More than once a week
- Once a week
- Every two weeks
- Once a month
- Every two months
- Less than every two months

What do / did you do during your time with your mentor? (tick all responses that apply)

- Sit and talk with them
- Home work / Education
- Leisure activity e.g. eating a meal out / cinema / sport etc
- Attend an appointment e.g. Doctor / Social Worker / Clinic
- Other (please specify)

How long will you / did you have a mentor for?

- Less than 6 months
- 6 months to 1 year
- 1 year to 1.5 years
- 1.5 years to 2 years
- More than 2 years

Do / did you enjoy having a mentor?

Y/N

Please explain what you like/d about having a mentor

……………………………………………………………………………………………………………………………………………………………………………………………………

Please explain what you do / did not like about having a mentor

……………………………………………………………………………………………………………………………………………………………………………………………………

If your mentoring has ended, how did you feel when it finished?

……………………………………………………………………………………………………………………………………………………………………………………………………

SECTION 3:

Have you ever been a mentor for someone else? Y/N

IF YES CONTINUE SECTION, IF NO SKIP TO SECTION 4

If you have mentored more than one person, please answer in relation to the person that you had most contact with.

Who is/was the person you mentored? The person I mentored was from ……..

(Please tick one)

- School / college
- Youth centre / Connexions
- Mental Health Service
- Religious organisation
- Youth Offending Team / Youth Justice Service
- In care
- Other (please specify) …………………………………

Is/was the person you mentored a male or a female?

Male / Female

How old is/was the person you mentored?

(please enter the actual or approximate age)

……….

Do/did you mentor them in a group setting or on a one-to-one basis?

(please choose one)

- Group
- One-to-one
- Both

How often do/did you meet with the person you mentored?

- More than once a week
- Once a week
- Every two weeks
- Once a month
- Every two months
- Less than every two months

What do/did you do during your time with the person you mentored?

(Tick all responses that apply)

- Sit and talk with them
- Home work / Education
- Leisure activity e.g. eating a meal out / cinema / sport etc
- Attend an appointment e.g. Doctor / Social Worker / Clinic
- Other (please specify)

How long will you be/were you a mentor for?

- Less than 6 months
- 6 months to 1 year
- 1 year to 1.5 years
- 1.5 years to 2 years
- More than 2 years

Do/did you enjoy being a mentor?

Y/N

Please explain what you like/d about being a mentor

……………………………………………………………………………………………………………………………………………………………………………………………………

Please explain what you do/did not like about being a mentor

……………………………………………………………………………………………………………………………………………………………………………………………………

SECTION 4:

Mentors in the Carmen Study are expected to meet with a young woman once a week, face to face, for one year.

In recognition of their time and effort, mentors receive £40 vouchers per month and they can gain a level 1 qualification called an ASDAN.

If the Carmen Study was available in your area, would you consider becoming a mentor for a young woman in care?

Please note: Yes / No

(If ticked Yes) What would be your reasons? (Please tick up to 3)

- Something to put on my CV
- Helping a young person in care
- Sharing my experiences
- New skills
- A recognised qualification
- Payment
- Other (please state) …………………………

(If ticked No) What would be your reasons? (Please tick up to 3)

- I am not interested in being a mentor for anyone
- I am not interested in being a mentor for a young woman in care
- I have issues to sort out in my own life
- I have too many education / employment commitments
- I have too many social commitments i.e. want my spare time for myself / friends / social or religious organisations
- I have family commitments e.g caring for children / family member
- I have no baby sitter for my children
- Other (please state) ……………………………………

SECTION 5:

Mentors in the Carmen Study volunteer to meet with the young woman they are mentoring once a week, face to face, for one year.

In recognition of their time and effort, mentors receive £40 vouchers per month and they can gain a level 1 qualification called an ASDAN.

On a scale of 1-5, how satisfactory are these incentives?

| Unsatisfactory Satisfactory |
| --- |
| 1 2 3 4 5 |

Please tell us what amount per month you thinks is satisfactory as a voucher payment in recognition of time and effort for mentors.

£…..

(This question is not to be seen by those who said they would never consider being a mentor) Would you consider becoming a mentor if there was no financial reward?

Y/N

Mentors in the Carmen Study have £15 a week to pay for an activity of their choice and travel costs for themselves and the young woman that they mentor.

On a scale of 1-5, how satisfactory is this amount for activities and travel?

| Unsatisfactory satisfactory |
| --- |
| 1 2 3 4 5 |

Please tell us what amount per week you think is satisfactory.

£…..

SECTION 6:

The training to become a mentor in the Carmen Study takes place over 3.5 days.

In recognition of their time and effort for attending the training, mentors receive a £30 voucher and reimbursement for travel expenses on completion of the training.

Do you think you would have any difficulties attending 3.5 days of training?

Y/N

If yes, what would the difficulties be? (choose up to 2 main difficulties)

- I have education / employment commitments
- I have social commitments i.e. want my spare time for myself / friends / social or religious organisations
- I have family commitments e.g caring for children / family member
- I have no baby sitter for my children
- Other (please state) …………………………………

On a scale of 1-5, how satisfactory is a £30 voucher and reimbursement of travel costs in recognition of the time and effort of attending training?

| Unsatisfactory Satisfactory |
| --- |
| 1 2 3 4 5 |

Please tell us what amount you think is satisfactory

………………………………………………………

(Question not for those that said they would never be mentor) Would you attend the training if there was no financial incentive on offer?

Y/N

When do you think is the best time for mentor training to take place?

- Weekdays
- Evenings
- Weekends
- A mixture of weekdays and weekends
- College / university holidays
- Don’t mind

SARAH – IF THEY PICK

1. Weekdays – Next question would be …..

If the training was delivered during weekdays, it would need to be completed within a one month period.

Would you like the training to be delivered over

1. four weekdays in a row
2. Two weekdays one week and two weekdays the following week
3. One weekday each week, delivered over 4 weeks
4. Other please state………………..
5. Evenings – Next question would be …..

If the training was delivered during the evenings it would be delivered over approximately 8 evenings. The training would need to be completed within a one month period.

Would you like the training to be delivered over

1. 4 evenings per week (training is completed over 2 weeks)
2. 3 evenings per week (training is completed over 3 weeks)
3. 2 evenings per week (training is completed over 4 weeks)
4. Weekends – next question would be……

If the training was delivered during weekends, it would need to be completed within a one month period.

Would you like the training be delivered over

1. Two weekends in a row (Saturday and Sunday)
2. Four weekends in a row (e.g -every Saturday for 4 weeks)
3. Other please state………………..
4. A mixture of weekdays and weekends – next question would be……

If the training was delivered during weekdays and weekends, it would need to be completed within a one month period.

Would you like the training be delivered over

1. One week and weekend (e.g Wed – Sat)
2. Two weeks and two weekends (e.g Fri and Sat for two weeks in a row)
3. One day a week over a month period (sometimes a weekday and sometimes a weekend day)
4. Other please state………………..

SECTION 7:

Mentors in the Carmen Study volunteer to meet with the young woman they mentor once a week, face to face, for one year.

Do you think once a week is: (please pick one)

- Too little
- Just right
- Too much

Do you think you would have any difficulties in meeting up with a young woman once a week?

Y/N

IF YES, what would those difficulties be? (tick up to 2 responses)

- I have education / employment commitments
- I have social commitments i.e. want my spare time for myself / friends / social or religious organisations
- I have family commitments e.g caring for children / family member
- I have no baby sitter for my children
- Other (please state) ……………………………………

How much time do you think is reasonable to expect a mentor to travel to see a young woman they are mentoring? (tick one)

- Less than 30 mins
- 30 mins
- 1hr
- 1.5 hrs
- As long as it takes as it doesn’t matter how far away they live

SECTION 8:

Now imagine you are mentoring a young woman in care who is aged 14-18…..

How often would you like to meet up with the young woman you are mentoring?

- Once a week
- Every two weeks
- Every three weeks
- Once a month
- Less than once a month

How long would you like to be in contact with the young woman you are mentoring?

- Less than 6 months
- 6 months to 1 year
- 1 year to 1.5 years
- 1.5 years to 2 years
- More than 2 years

You have got to know the young woman you are mentoring.

Do you think you would you feel comfortable discussing their sexual relationships and sexual health with them?

Y/N

What type of support do you think should be available to you whilst you are a mentor? (Tick up to 3)

- Phone contact with a project coordinator from the Local Authority who manages the mentoring and provides support to mentors
- Individual, face to face support with a project coordinator from the Local Authority
- A group session which includes the other mentors, facilitated by project coordinator from the Local Authority
- Individual (face to face or phone) support from a professional that I know already
- Other (please state) ………………………………………………………….

The next page should list the options above that they have ticked and a box where they need to put how often they want that type of support.

Options will be:

More than once a week

Once a week

Every two weeks

Monthly

Every two months

Mentors in the Carmen Study have monthly support group meetings with other mentors, facilitated by a project coordinator. These provide an opportunity to discuss with other mentors and the project coordinator how things are going and they try to resolve any issues.

What kind of professional do you think should facilitate these meetings and provide support to mentors? (i.e take on the role of project coordinator)

- Social worker
- Local Authority Health professional (including sexual health workers)
- Other Local Authority professional (e.g. personal advisor / participation officer/youth worker)
- Other professional who is independent from Local Authority e.g. Charity / voluntary organisation worker
- A mentor who has graduated the mentoring programme
- Other please state ………………………………………………………..

SECTION 9

Imagine you see a poster about the Carmen Study with details about the study and the contact details for the project coordinator.

You are interested in taking part. Would you feel able to call the project coordinator?

Y/N/ not sure?

How would you prefer to be first contacted with information about the Carmen study?

- In person
- Phone
- Email
- Post
- social network site / twitter
- Other (please state) ………………………

Who would you prefer to first contact you about the Carmen study? (choose one)

- My social worker or a previous social worker
- Another professional that I have contact with e.g. tutor / personal advisor
- A researcher
- The Carmen study project coordinator (even if I don’t know them)
- A Carmen Study mentor
- A key worker / carer / family member
- It don’t mind who I receive initial information from
- Other (please state)

We would like more ideas about where to advertise the Carmen Study. Please tell us about any other places female care leavers (age 19-25) might visit where someone could let them know about the study or the Carmen Study could put up posters ………………………………………………………………………………………….

………………………………………………………………………………………….

Thank you for completing the Carmen Research Study Questionnaire.

By completing this survey you have helped us to understand a bit more about what young women like you think and feel about the research and peer mentoring.

If you would like to be entered into the Prize Draw to win £30 in Love2Shop Vouchers, please complete your name and contact details below. The winner will be randomly drawn from completed questionnaire entries on or soon after the 14^th^ December 2012 and will be contacted straight away.

Name

Email (Essential so we can email to let you know if you have won)

Phone (not essential)

This personal information will be stored separately from your responses so that your answers remain anonymous.

If you have any questions, you can contact us on [carmen@sgul.ac.uk](mailto:carmen@sgul.ac.uk)

Or:

Deborah Meyer, Research Trial Manager on 02087255523

Fiona Clare, Research Assistant on 02087252625
